# Genetic Diversity of Rotavirus Strains Circulating in Environmental Water and Bivalve Shellfish in Thailand

**DOI:** 10.3390/ijerph110201299

**Published:** 2014-01-24

**Authors:** Leera Kittigul, Apinya Panjangampatthana, Kitwadee Rupprom, Kannika Pombubpa

**Affiliations:** Department of Microbiology, Faculty of Public Health, Mahidol University, 420/1 Ratchawithi Road, Bangkok 10400, Thailand; E-Mails: pin_pooh@hotmail.com (A.P.); kitwadee.rp@gmail.com (K.R.); kannika.pom@mahidol.ac.th (K.P.)

**Keywords:** rotavirus, genotype, water, oyster

## Abstract

Rotavirus is a common cause of acute diarrhea in young children worldwide. This study investigated the prevalence and molecular characterization of rotavirus in environmental water and oyster samples in Thailand. A total of 114 water samples and 110 oyster samples were collected and tested for group A rotavirus using RT-nested PCR. Rotavirus genotype was identified by phylogenetic analysis of the VP7 genetic sequences. Group A rotavirus was detected in 21 water samples (18.4%) and six oyster samples (5.4%). Twenty five rotavirus strains were successfully sequenced and classified into four genotypes; G1, G2, G3, and G9. Rotavirus G1 (three strains), G2 (three strains), and G9 (two strains) demonstrated the genetic sequences similar to human strains (90%–99% nucleotide identity), whereas G3 (17 strains) was closely related to animal strains (84%–98% nucleotide identity). G1 strains belonged to lineages I (sub-lineage c) and II. G2 strains belonged to lineage II. G9 strains belonged to lineages III (sub-lineage b) and IV. G3 strains belonged to lineages I, III (sub-lineage c), and IV with a predominance of lineage I. The present study provides important information on the rotavirus strains circulating in the environment.

## 1. Introduction

Rotaviruses are the leading cause of acute diarrhea worldwide; mainly in infants and young children aged <5 years. An estimated 453,000 children, in this age group, died from rotaviruses in 2008 [1]. Among those countries participating in the global surveillance network for rotaviruses in 2009, 36% of hospitalized children aged <5 years with acute diarrhea had a rotavirus infection [2]. Rotaviruses, belonging to the genus *Rotavirus* in the family *Reoviridae*, contain 11 segments of double-stranded RNA surrounded by capsid proteins without an envelope [3]. Based on antigenic and genetic properties, eight groups (A to H) of rotaviruses have been classified and group A rotaviruses are the most common cause of acute gastroenteritis in humans [4]. There are at least 27 G genotypes and 37 P genotypes of rotaviruses identified in humans and animals [5,6]. The most prevalent rotavirus strains recovered from infected patients are G1P[8], G2P[4], G3P[8], G4P[8] and G9P[8] (see reference [7]). 

Rotaviruses are transmitted primarily via fecal-oral route, through person-to-person contact and are shed in high concentrations. The viruses are also transmitted via fecally contaminated food, water and environmental surfaces [3]. Water- and shellfish-borne outbreaks, caused by rotaviruses, have been reported, possibly associated with sewage contamination [8,9,10,11]. Surveillance studies monitoring rotaviruses confirm the circulation of rotaviruses in bivalve shellfish and aquatic environments [12,13,14,15,16] indicating the potential for rotavirus transmission to humans. Molecular characterization of rotaviruses could contribute epidemiological information for the implementation of an effective vaccine. Although two licensed rotavirus vaccines (Rotarix: GlaxoSmith-Kline Biologicals, Rixensart, Belgium and RotaTeq: Merck & Co., Inc., West Point, PA, USA) have been available for use in Thailand, they are not yet included as part of the national immunization program. Extensive studies of rotavirus strains in patients with acute gastroenteritis have been documented [17,18,19,20]. However, the data on rotavirus genotypes in the environment are limited. Using hospital-based studies, the highest prevalence rate of rotavirus gastroenteritis is found in children <5 years of age [21,22,23] and rotavirus infection also occurs in adult patients with acute gastroenteritis in Thailand [24]. This study aimed to determine the presence of rotaviruses and to characterize rotavirus genotypes in both water and bivalve shellfish samples collected in Thailand. The genetic variation and genetic relationship, between rotavirus strains in the present study and reference rotavirus strains available in the public database, were also analyzed for human and animal rotaviruses. 

## 2. Experimental Section

### 2.1. Water and Oyster Samples

A total of 114 environmental water samples, including 59 from the Lop Buri river and 55 from irrigation canals were collected monthly at different collection sites in Lop Buri Province, Thailand from September 2006 to January 2007. The Lop Buri Province, located in the central region, is 155 km from Bangkok, the capital city. The Lop Buri river is about 95 km long passing through the town of Lop Buri. Irrigation canals are permanent constructed channels, about 134 km long, flowing through the Province. The Lop Buri river and irrigation canals split off from the Chao Phraya River which is a major river in Thailand. Water collection sites were approximately 500 m apart. For each sample, 1 L of water was collected from a depth of at least 30 cm below the water surface, kept in a chilled container, and transported immediately to the laboratory. The temperature and pH of the water samples ranged between 23–33 °C and 6.0–8.6, respectively. 

Between April 2008 and March 2009, 110 oyster samples (*Crassostrea belcheri*) were collected from harvesting areas in Surat Thani Province, located in the south, 685 km from Bangkok. All oyster samples were transported to the laboratory in chilled containers. Immediately after arrival, the oysters were washed, scrubbed, and shucked aseptically. The digestive tissues, from a pool of three oysters/sample, were dissected and 4 g weighed. All water and shellfish samples were processed in order to concentrate the viruses. 

### 2.2. Virus Processing

#### 2.2.1. Water Samples

All water samples were concentrated using a modified previously described adsorption-elution technique [25]. Briefly, 1 L of water samples were adjusted to pH 3.5 with 1 N HCl, and aluminum chloride was added for a final concentration of 0.0015 N to flocculate the virus. Then, the water samples were stirred at room temperature for at least 30 min and passed through membrane filters which were 47 mm in diameter with a 0.45 µm pore size (Gelman, Ann Arbor, MI, USA). After washing the membranes with 0.14 M NaCl, pH 3.5, the virus was eluted by adding a 2.9% tryptose phosphate broth (TPB) containing 6% glycine, pH 9.0 (5 mL/membrane filter: 2–4 filters/sample). The eluates (10–20 mL) were adjusted to pH 7.0–7.4 with 4 N HCl and reconcentrated using a centrifuge with aspirator vacuum pump (UNIEQUIP Laborgeratebau und-vertriebs GmbH, Munich, Germany) at 3 °C for approximately 5 h. The aspirator pump system (UNIJET II) used built-in refrigeration to continuously chill the water reservoir to counter the normal heat build-up from the pump action. Cold water, from which the air was evacuated, from the sample chamber and the eluates were evaporated gently. Volumes of the concentrates were reduced to1.5–4.2 mL. The concentrates were stored at −80 °C until used for nucleic acid extraction.

#### 2.2.2. Oyster Samples

Digestive tissues from oyster samples were concentrated using the previously described adsorption-elution-concentration technique [26]. Briefly, chilled, sterilized distilled water (60 mL) was added to 4 g of digestive tissues and homogenized by a blender at high speed. The homogenate was adjusted to pH 4.8, shaken for 15 min, and centrifuged at 3,000 × g for 15 min at 4 °C. The supernatant was discarded and the pellet was suspended in 4 mL of 2.9% TPB, containing 6% glycine, pH 9.0, for elution of the virus, shaken at 215 rpm for 15 min on ice, and centrifuged at 10,000 × g for 15 min at 4 °C. The supernatant (S1) was collected and the pellet was re-eluted with 4 mL of 0.5 M arginine-0.15 M NaCl, pH 7.5. The suspension was shaken at 230 rpm for 15 min on ice and centrifuged at 10,000 × g for 15 min at 4 °C. The supernatant (S2) was collected, combined with S1 and adjusted to pH 7.5. The virus in the supernatant was precipitated by adding 12.5% PEG 8,000 and 1.9% NaCl (PEG-NaCl). The mixture was stirred for 2 h, refrigerated overnight, and then centrifuged at 10,000 × g for 1 h at 4 °C. The pellet was dissolved in 6 mL of 0.05 M phosphate-buffered saline (PBS), pH 7.5 and precipitated again with PEG-NaCl. The mixture was stirred for 2 hr and then centrifuged at 10,000 × g for 10 min at 4 °C. The pellet was dissolved in 3 mL of 0.05 M PBS. The virus was extracted with chloroform at a final concentration of 30%. After centrifugation at 3,000 × g for 10 min, the top layer of the aqueous phase was collected (A1). The pellet was re-extracted with 0.5 volumes of arginine-NaCl, pH 7.5. After centrifugation, the top layer (A2) was collected and combined with A1. The sample was re-concentrated using a vacuum centrifuge to reduce the volume of the concentrate to approximately 1 mL and stored at −80 °C until nucleic acid extraction. 

### 2.3. RNA Extraction and RT-nested PCR

A 140 μL aliquot of water concentrate was extracted for viral RNA using the QIAamp^®^ Viral RNA extraction kit (QIAGEN GmbH, Hilden, Germany) and 60 μL of the RNA extract was collected. A 200 μL aliquot of oyster concentrate was extracted for viral RNA using the RNeasy^®^ mini kit (QIAGEN GmbH) and a 50–55 μL aliquot of the RNA extract was collected.

Rotavirus RNA was detected using the previously described RT-nested PCR method [26]. The extracted RNA (2 μL) was heated at 94 °C for 4 min and placed on ice for at least 10 min. RNA was examined using the SuperScript^TM^ One-Step RT-PCR system with Platinum^®^*Taq* DNA polymerase (Invitrogen, Life Technologies, Carlsbad, CA, USA). One-Step RT-PCR was performed with a 50 µL reaction volume. The extracted RNA sample was added to the RT-PCR mixture (48 μL) consisting of 1X Reaction Mix (a buffer containing 0.2 mM of each dNTP, 2 mM MgSO_4_), SuperScript^TM^ III RT/Platinum^®^*Taq* Mix, 0.25 µM primer RV1, 0.25 µM primer RV2 [27] and nuclease-free water. The RT and PCR were carried out with following steps: RT at 41 °C for 60 min; PCR cycle 1–25, 94 °C for 2 min, 94 °C for 30 sec, 55 °C for 30 sec, 72 °C for 60 sec with the final extension of 72 °C for 3 min. For nested PCR, a 1 μL of the RT-PCR product was further amplified under the same conditions of amplification as for the first PCR, except for changing the primer pair to RV3 and RV4 [27] and their concentrations to 0.5 μM and the concentration of MgCl_2_ to 3.5 mM. PCR products were analyzed by 1.5% agarose gel electrophoresis and ethidium bromide staining. A DNA fragment of 346 bp was considered to be the rotavirus DNA.

### 2.4. Phylogenetic Analysis of Rotavirus-positive Samples

The genetic characterization of group A rotaviruses was performed by sequencing and phylogenetic analysis of the nested PCR amplicons. Amplified products (346 bp) were purified using the QIAquick PCR purification kit (QIAGEN GmbH) and sequenced at the Bioservice Unit of the National Science and Technology Development Agency (Bangkok, Thailand) using the same forward (RV3) primer. The nucleotide sequences of VP7 gene were compared with those of the reference strains available in the NCBI (National Center for Biotechnology Information) GenBank database using BLAST (Basic Local Alignment Search Tool) server [28]. Phylogenetic and molecular evolutionary analyses were conducted using MEGA, version 5.1 [29].

The nucleotide sequences of rotavirus detected in water and oyster samples, corresponding to fragments of the VP7 gene of rotavirus, were deposited in GenBank under the accession numbers KF907102–KF907126.

## 3. Results

### 3.1. Rotavirus Detection in Environmental Samples

Based on the RT-nested PCR, rotaviruses were found in 18.4% (21 of 114) of the water samples collected. The prevalence of rotaviruses in a river (27.1%) was approximately three times higher than that in irrigation canals (9.1%). Regarding the oyster samples collected, rotaviruses were found in 5.4% (6 of 110), as shown in Table 1. Rotaviruses were detected in the water samples collected in October, December, and January and in the oyster samples collected in November, December, January, and March (data not shown).

**Table 1:** Group A rotavirus in environmental water and oyster samples detected by RT-nested PCR.

Source of Samples	No. of Samples	No. of Rotavirus-positive Samples (%)
River	59	16 (27.1)
Irrigation canals	55	5 (9.1)
Oysters	110	6 (5.4)
Total	224	27 (12.0)

### 3.2. Molecular Analysis of Rotaviruses

All 21 water samples (100%) and four (66.7%) oyster samples positive for group A rotavirus were successfully sequenced and analyzed. Using the BLAST program, rotavirus strains detected in river, irrigation canal and oyster samples revealed similar nucleotide sequences to human rotaviruses (90%–99% nucleotide identity) in eight samples and animal rotaviruses (84%–98% nucleotide identity) including canine, caprine, and swine rotaviruses in 17 samples.

**Figure 1:**
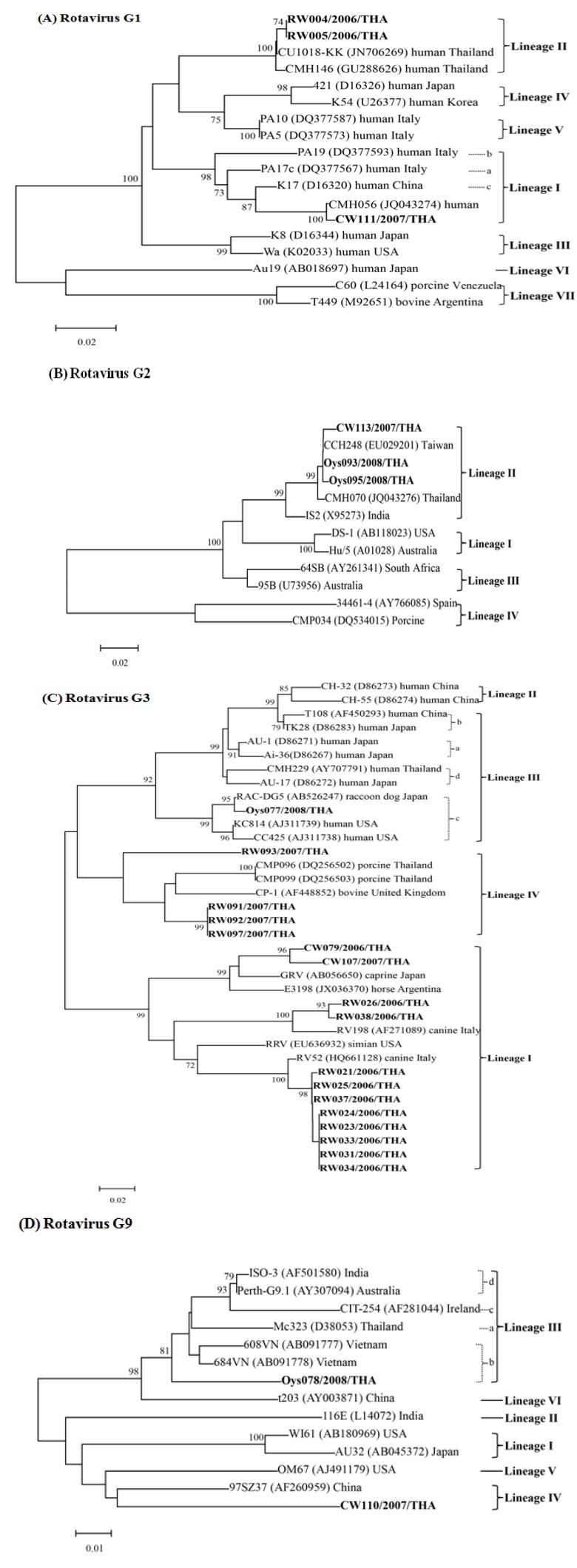
Phylogenetic trees of partial VP7 nucleotide sequences derived from rotavirus strains detected in this study and other reference strains from the GenBank database. A, B, C, and D represent G1, G2, G3, and G9 genotypes, respectively. The trees were generated using the neighbor-joining analysis of 1000 repetitions in MEGA 5.1. The scale bar indicates nucleotide substitutions per site. Bootstrap values >70% are shown at the branch nodes. Rotavirus strains in the present study are indicated in bold.

By phylogenetic analysis of the partial VP7 nucleotide sequences for lineage classification by Chaimongkol *et al.* [30] and Than *et al.* [31], the 25 rotavirus strains could be classified into four genotypes; G1, G2, G3, and G9. Within G1 (Figure 1A), three strains clustered into two different lineages. One strain (CW111), from an irrigation canal, belonged to sub-lineage c of lineage I and showed a 98% nucleotide identity with a Thai strain found in humans (CMH056; accession number EF199712). Two strains (RW004 and RW005) from a river were also closely related to the Thai strain found in humans (CU1018-KK; JN706269) and belonged to lineage II with 96% and 97% nucleotide identity. One strain, from an irrigation canal (CW113), and two strains, from oysters (Oys093 and Oys095), belonged to lineage II of G2 (Figure 1B) with 97%–99% nucleotide identity to human strains occurred in both Taiwan (CCH248; EU029201) and Thailand (CMH070; JQ043276). 

For G3 (Figure 1C), 12 rotavirus strains clustered in distinct branches of lineage I. Eight strains (RW021–RW037), and two strains (RW026 and RW038) from a river, were closely related to the canine strains RV52 (HQ661128) with 94%–98% nucleotide identity and RV198 (AF271089) with 93% and 96% nucleotide identity found in Italy, respectively. Two strains from irrigation canals (CW079 and CW107) were genetically similar to the caprine strain GRV (AB056650) with 91% and 95% nucleotide identity. One strain from oyster (Oys077) was closely related to the rotavirus strain (RAC-DG5; AB526247) isolated from a raccoon dog in Japan with a 98% nucleotide identity and belonged to lineage III (sub-lineage c). In addition, the other four strains of G3, from a river, belonged to lineage IV and were similar to the genetic sequences of porcine strains (CMP096; DQ256502 and CMP099; DQ256503) in Thailand. G3 strains in the river samples (RW091, RW092, and RW097) showed a 91%–93% nucleotide identity. Of note, one G3 (RW093) exhibited an 84% nucleotide identity lower than the other G3 (lineage IV) strains. In the G9 tree (Figure 1D), two strains clustered in different branches. One strain (Oys078), from oysters, was closely related to 608VN (AB091777) in Vietnam with a 95% nucleotide identity and belonged to lineage III (sub-lineage b), whereas the other strain (CW110) from an irrigation canal belonged to lineage IV and was genetically similar to the strain in China (97SZ37; AF260959) with a 90% nucleotide identity**.**

VP7 nucleotide sequences of these rotavirus strains detected in water and oyster samples were compared with rotavirus vaccine strains. G1 strains, from the river (RW004 and RW005) and irrigation canal (CW111), shared 91%–96% and 89%–91% nucleotide identities with the VP7 of the G1 strain Rotarix (A41CB052A; JN849114) and RotaTeq (WI79-9; GU565057), respectively. G2 strains, from the irrigation canal (CW113) and oyster (Oys093 and Oys095) were related (92–93%) to VP7 of the G2 strain (SC2-9; GU565068) of RotaTeq. G3 animal strains from the river (RW021–RW097) and irrigation canals (CW079 and CW107) were less related (78%–83%), whereas the strain from oyster (Oys077) was more related (89%) to VP7 of the G3 strain of RotaTeq (WI78-8; GU565079).

## 4. Discussion

In this study, an adsorption-elution technique and RT-nested PCR previously developed in our laboratory [25] were used to examine the rotavirus in environmental water and oyster samples. The method could detect rotaviruses at the lowest concentration of 5 × 10^2^ plaque forming units (PFU)/1 L water and 125 PFU/g oyster. In seeding experiments of rotavirus in water, no rotavirus was lost during the concentration process [32], however, virus loss was observed at the concentration steps for spiking rotavirus in oysters [26].

Rotavirus was found in the water samples at a higher frequency than the oyster samples. The results of the higher detection rate of rotavirus in river samples than in the irrigation canal samples suggest that the river water was more polluted with human and/or animal fecal excreta than irrigation canal water. The river passes through both urban and rural areas and has been consequently impacted by a continuous discharge of domestic sewage and animal waste, whereas irrigation or water supply canals are used for the conveyance and delivery of water for municipal uses and agriculture irrigation. The prevalence rate of rotavirus in river samples from Thailand is higher than that in surface water samples from Slovenia [33]. This may be due to the differences of sample type, collection site and detection methods. High detection rates of rotavirus in water samples have been reported from poor sanitation environments in Brazil of 88% [16] and Uganda of 60.9% [34]. 

The detection rate of rotavirus in oyster samples from this survey study is comparable to the previous study in Thailand of 3.3% [26]. In France, oyster samples were more highly contaminated with rotavirus at a frequency of 27% because the oysters were collected in areas containing human sewage [35]. It is likely that the oyster samples in the present study were collected from the oyster farms where were contaminated with low level of human excreta. Both water and oyster samples contaminated with rotavirus were collected mostly in the cool weather (winter season) of Thailand, corresponding to the period of a high prevalence of rotavirus in hospitalized patients [24]. The presence of rotavirus in water sources may cause the virus to spread and be a potential vehicle for virus transmission leading to rotavirus outbreaks [9,10,11]. In addition, bivalve shellfish, such as oysters and mussels, can accumulate rotavirus [15,17] and might be associated with a gastroenteritis outbreak [8]. 

All four genotypes (G1, G2, G3, and G9) of rotavirus strains are present in irrigation canal water, three genotypes (G2, G3, and G9) in oysters, and two genotypes (G1, and G3) in river water. A number of rotavirus strains are similar to human strains; G1, G2, and G9. These rotavirus genotypes found in the environmental samples are in accordance with rotavirus genotypes detected in fecal specimens of patients with acute gastroenteritis from the same area of Thailand during the same period [36]. The findings support the circulation of rotavirus genotypes both in clinical specimens and environmental samples. 

Although the distribution of rotavirus genotypes has changed over time and varies in different geographical areas; rotavirus G1 continues to be the most common genotype in humans [30,37]. Rotavirus G1 strains detected in river water (lineage II) are different from G1 in irrigation canal water (lineage I, sub-lineage c) but the G1 strains in both water sources are close to the human strains reported previously in Thailand [30]. Rotavirus G2 is the second common genotype among rotavirus strains reported worldwide. Lineage II found in canal water and oyster have been considered to be spread by patients with rotavirus infection in Asian countries [30]. Although an irrigation canal water and oyster samples were collected from different regions in Thailand, rotavirus strains detected were in the same lineage showing a close genetic relationship to G2. 

Of particular interest is the great number of rotavirus G3 strains detected in all three sample types, especially in river water, which are related to animal strains. This finding indicates that the river is a fecal source of domestic animals. G3 strains (lineage I) from river samples had a close genetic relationship with the two canine rotavirus strains (RV 52-96 and RV 198/95) from Italy. Re-assortment and interspecies transmission events have been suggested as occurring among these two canine strains, feline, and human rotaviruses and contributing to the genetic diversity [38]. The lineage III (sub-lineage c), showing a different branch in the phylogenetic tree from other human strains, is associated with an animal strain (raccoon dog). Lineage IV sequences similar to the porcine rotavirus strains are found in diarrheic piglets in Thailand [39]. Lineage III (sub-lineage d) of rotavirus G9 is predominant, as reported around the world [31], but, as this study shows, a different sub-lineage (sub-lineage b) in oyster and also lineage IV in canal water. Taken together, rotavirus strains detected in the environment demonstrate the genetic diversity of human and animal rotavirus strains with different genotypes, lineages, and sub-lineages.

For VP7 lineage, the G1 genotypes of Rotarix and Rotateq vaccine strains are present in lineages II and III, respectively. The G2 and G3 strains of RotaTeq belong to lineage II [40]. In the present study, although G1 strains were in the same lineage as the strain of Rotarix and also G2 strains were in the same lineage as that of RotaTeq, the VP7 nucleotide sequences of rotavirus strains in water and oyster displayed relative differences with the VP7 of the G1 strain in Rotarix, and G1 and G2 strains in RotaTeq. G3 strains found in the environment are all animal strains with different lineages from the G3 strain in RotaTeq. It seems that the circulating strains are naturally occurring rotaviruses possibly shed by humans and animals.

## 5. Conclusions

The present study highlights the genetic variation in the VP7 gene among rotavirus strains circulating in the environment. This study is the first to provide extensive data for rotavirus strains detected in water and bivalve shellfish samples. The findings indicate the existing of G1, G2, G3, and G9 rotavirus genotypes with different lineages. The information provided on the genetic relatedness of different rotavirus strains will be useful for molecular epidemiological study and the study of virus evolution.
